# Assessing technical aspects and vulnerabilities from Tswaing informal settlement in Hammanskraal, South Africa

**DOI:** 10.4102/jamba.v17i1.1890

**Published:** 2025-07-18

**Authors:** Davies V. Nkosi, Patience Mbola, Evelyn R. Maleka, Dikeledi M. Mapheto, Engetelo P. Ngwenya, Thabang S. Mokwena, Silas Mohlala, Nchang J. Langa, Lethabo S. Kgopa, Octavia M. Komane

**Affiliations:** 1 Department of Environmental Health, Faculty of Science, Tshwane University of Technology, Pretoria, South Africa; 2 Emergency Services, Department of Disaster Risk Management, City of Tshwane Metropolitan Municipality, Pretoria South Africa

**Keywords:** hazards, vulnerabilities, environmental health, emergencies, community resilience, resources

## Abstract

The increasing frequency of hazards such as floods, droughts, socio-political unrest, emerging diseases and rising temperatures has affected South Africa. In line with the Sendai Framework Principles (2015–2030), which anticipate that these hazards can deplete local resources and lead to disaster declarations, this empirical study investigated community vulnerabilities in Tswaing informal settlement during South Africa’s dry winter season. Researchers collaborated with community leaders and officials from the City of Tshwane Metropolitan Municipality to gain access to the study area. Using vulnerability capitals, hazard indicators were systematically identified and recorded according to a standard vulnerability assessment formula. Seven groups of students from the Environmental Health Programme at Tshwane University of Technology undertook transit walks throughout the settlement. RG101 drones equipped with Global Positioning System and smartphone connectivity were utilised for imaging and to survey inaccessible areas.

**Contribution:**

The vulnerability levels of each capital, along with corresponding indicators and trigger points observed during the transit walks, were calculated. The results indicated that while the community demonstrates a degree of resilience and adaptive capacity, several critical weaknesses remain. These include prevalent illegal electricity connections and poor waste and water quality management. The study highlights the significant role of public participation in hazard management, emphasising the importance of community engagement to ensure water quality management, basic hygiene and electricity safety awareness. These participatory measures are essential for raising awareness about potential hazards and reducing the risk of subsequent disasters.

## Introduction

Emergency responses as a service in a community that has been hit by hazards are generally embedded in stage number 3 of the disaster management circle. Many disaster management articles emphasise the importance of ensuring that life continues as normal and that the general impact of the hazard is cushioned against causing unnecessary community effects (Hou et al. 2025). In many cases, a hazard would have already been identified in a community; this stage provides an opportune time to create responses that emphasise the importance of preparing for the worst (Hou et al. 2025). This article acknowledges this fact and aims to map out and emphasise the role and importance of a pre-assessment of communities, terming this intervention ‘a vulnerability assessment’ (Mubarak, Amiruddin & Gaus 2019). In many instances, ‘vulnerability and capacity assessments’ (VCA) are described as intervention processes that seek to raise awareness and estimate the level of harm that could be experienced by a community (Berse 2018; Morgan 2011).

To further unpack this concept of VCA, Sahar et al. (2017) emphasises that it is a process of engaging the community, current local dynamics and literature to plan for the anticipation of the next disaster pressure point, rather than a cost assessment generally done after impact. In line with many assessments, Sahar et al. (2017) and Weichselgartner (2001) further suggest that VCA is generally performed when there is calm in a community and the anticipation of hazards is not even a priority to a community. In this approach, each suspected hazard could be identified and a response strategy formulated in anticipation of its occurrence (Flax, Jackson & Stein 2002; Sahar et al. 2017).

To shed light on this effect, it is important to note that disaster management and the key indicators for all of the response trajectories are intended to meet the emergency response-related needs (Zack 2023). Key performance areas, in the light of this knowledge, remain imperative; new and dynamic processes of disaster responses for specific hazards must be catered to before and after their occurrence while incorporating past experience and existing knowledge of coping with a hazard (Imperiale & Vanclay 2016). As part of awareness campaigns, it is important that the communities and stakeholders, such as environmental health, are empowered with specific skills to deal with emergency situations that may arise as a result of a hazard (Sahar et al. 2017; South Africa 2002).

Considering that environmental health is one of the responders in most emergency situations, they are generally tasked with the ability to identify community vulnerabilities, basic necessities for public health response and the pressure and release of hazards within the affected community (Mbola, Nkosi & Morakinyo 2024). As part of their training, students enrolled in environmental health must conduct a community assessment to identify and evaluate the existing vulnerabilities, their coping strategies and the possible adoption of mitigation strategies (Imperiale & Vanclay 2016). As part of the anticipation, it is imperative to note that specific early alert systems must have been developed. These alerts should provide insight into the type of hazard, its severity and a strategic view of the response plans (Comes, Mayag & Negre 2014).

The underlying ideology is the fundamental shift between the Hyogo Framework 2005–2015, which looked at understanding disasters from the perspective of the affected community and the Sendai Framework 2015–2030 that looked at the reduction of disaster impacts (Gautam & Khanal 2009; Hung et al. 2021; UNISDR 2005). This is clarified by the need to align with sustainable development goals 11 and 13 that clarify the need to reduce the number of people affected by disasters and the creation of resilient communities (Cabello et al. 2021; United Nations 2025). It is then important for emergency responders to be well capacitated as part of their training at the institutions of higher learning or any other relevant programmes (Imperiale & Vanclay 2016).

## Research methods and design

To provide practicality in this regard, an empirical case study of vulnerability assessment was conducted in Tswaing Settlement in the northern region of Gauteng province in Hammanskraal. This case study follows the approved protocol of an investigation cleared by the Tshwane University of Technology’s (TUT) Research Ethics Committee for a doctorate study in Environmental Health Disaster Management. The case study provides a snapshot view of dynamics faced by a typical informal settlement during the end of winter, which is predominantly a dry season of the year in South Africa. The region is classified as a Cwa climate where the temperatures are generally mild with dry winters and hot summers (Skybrary 2025).

To estimate community vulnerabilities, an integration of technology (YLR/C Drones and GPS Carmin eTrex 10 handheld navigators) was used during the VCA of Tswaing informal settlement. The surveillances were conducted by students enrolled in environmental health degrees as part of their disaster management work-integrated learning. The conditions were close to the normal operation of the community during the investigation period. This formed part of the VCA that followed a specific formula as clarified and predominantly used in community assessments (Usman Kaoje et al. 2021):

Guided by the nine vulnerability assessment capitals, students identified possible hazard trigger points (indicators) in the Tswaing informal settlement and recorded their occurrences.

### The study area

Tswaing, which means ‘place of salt’ in Tswana, is an area at the northeast of Pretoria near Hammanskraal with GPS coordinates location 25°25’09.07”S and 28°07’07.11”E. This is an informal settlement with a total number of between 300 and 500 households with an estimated population of ±2200 people (Statistics S A 2022). The area is an informal settlement, and as a result, there are no formal service delivery systems by the City of Tshwane Metro municipality.

### Data collection

**FIGURE 1:**
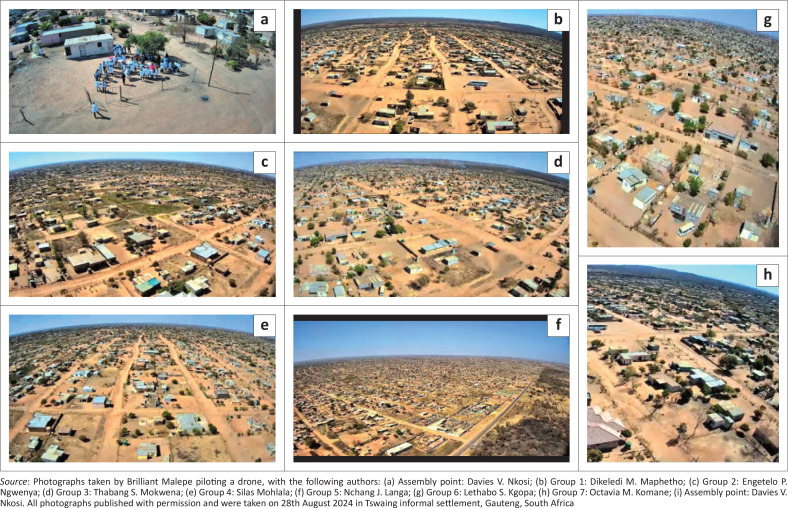
Group allocation for transit walks in Tswaing informal settlement: (a) Assembly point (b) Group 1 (c) Group 2 (d) Group 3 (e) Group 4 (f) Group 5 (g) Group 6 (h) Group 7.

**FIGURE 2:**
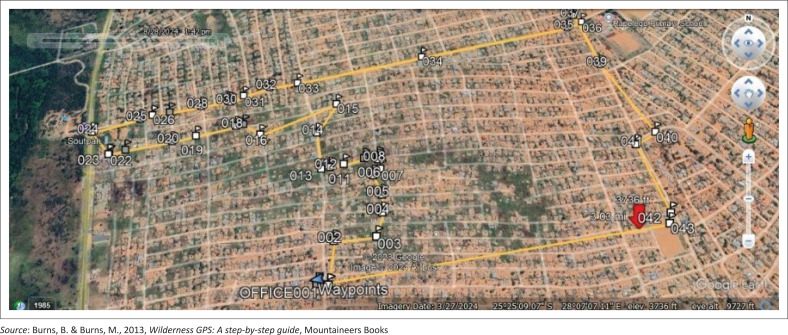
An example of global positioning system waypoints (trigger points) and transit walk of group 5.

**FIGURE 3:**
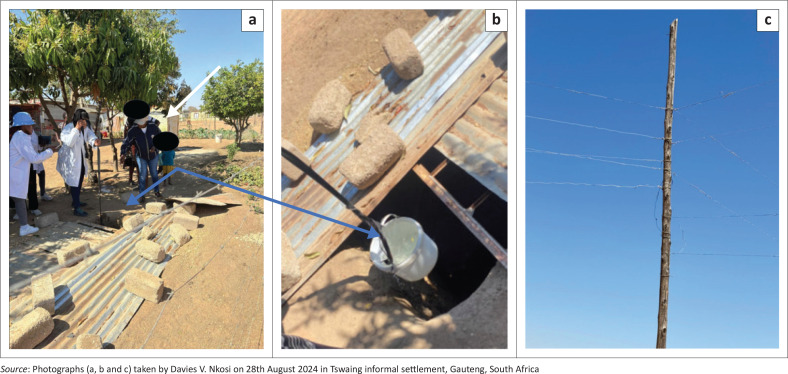
(a and b) Borehole and toilets proximities (c) Illegal electricity connection.

As part of data collection, students from TUT doing the 4th level of the degree Bachelors of Environmental Health were grouped in seven groups; each group comprising between 6 and 9 members was allocated areas for empirical data collection within different parts of the community (Figure 1, Figure 2, Figure 3a and 3b). Each group was assigned a minimum of three community leaders and a student drone pilot during the transit walk within the community. These leaders provided the students’ groups with community information where needed. Using a template of technical aspects identified by the World Health Organization’s Sphere project on humanitarian response (2018), students conducted transit walks within sections of the community. Different points of interest to environmental health were identified using a matrix prescribed by Whitney (2014) when investigating VCA. The vulnerability assessments of each specific capital with its indicator and trigger points as identified during the transit walk were calculated, and the possible indicators or results are presented in Table 1.

**TABLE 1:** Assessing community technical aspects and its vulnerability to specific indications.

Community vulnerability indicators	Group 1	Group 2	Group 3	Group 4	Group 5	Group 6	Group 7
**Social capitals**							
Level of education	√	√	√	√	√	√	√
Family networks	n/a	n/a	n/a	n/a	n/a	n/a	n/a
Low level of poverty	-	-	-	-	-	-	-
Inequalities	√	√	√	√	-	√	√
**Human capitals**							
Community skills	n/a	n/a	n/a	n/a	n/a	n/a	n/a
Age of community	√	√	√	√	√	√	√
Gender of the community	-	-	-	-	-	-	-
Knowledge and experience	n/a	n/a	n/a	n/a	n/a	n/a	n/a
**Economic capitals**							
Income levels	-	-	-	-	-	√	√
Job availability	-	-	-	-	-	-	-
Markets and trading abilities	-	-	-	-	-	-	-
Entrepreneurship	-	-	-	-	-	-	-
Agriculture	√	√	√	√	√	√	√
**Cultural capitals**							
Belief systems	-	-	-	-	-	-	-
Relativism	-	-	-	-	-	-	-
Norms and values	-	-	-	-	-	-	-
Biculturalism	√	√	√	-	-	-	-
**Natural ecology capitals**							
Land scape	√	√	√	√	√	√	√
Land use and management	-	-	-	-	√	-	-
Natural resources	-	-	-	-	-	-	-
Availability of water	-	-	-	-	-	-	-
Sanitation	√	√	√	√	√	√	√
**Infrastructure capitals**							
Roads and linkages	√	√	√	√	√	√	√
Waste management	-	-	-	-	-	-	-
Storm water drainages	-	-	-	-	-	-	-
Natural resources protection	-	-	-	-	-	√	-
Energy and power resources	-	-	-	-	-	-	-
Adaptable buildings	-	-	-	√	√	√	-
**Political capitals**							
Political leadership	√	√	√	√	√	√	√
Community governess	√	√	√	√	√	√	√
Rule of laws	√	√	√	√	√	√	√
**Institutional capitals**							
Police and advocacy	√	√	-	-	-	-	-
Hospitals and health care	-	-	-	-	-	-	-
Churches and religious structures	-	√	-	-	-	-	-
**Technological capitals**							
Community communications	√	√	√	√	√	√	√
Broadcast channels	-	-	-	-	-	-	-
Internet and access	√	√	√	√	√	√	√
Print media	-	-	-	-	-	-	-

√, Observable; n/a, Not observed or measured; -, non-existent in terms of group ratings.

Formula used to estimate the vulnerabilities of Tswaing settlement to specific hazards based on their indication (Whitney 2014), refer to equation 1 below:


$$v_{A}=\frac{H\times v}{C}$$ [Eqn 1]

where:

vA, vulnerability assessment,

H, hazard,

V, Vulnerability,

C, consequences.

To maximise the quality of drones’ image capturing, specific calibrations were carried out on the device, and the competence was established during the basic drone operations training. Basic steps of operations were followed as pointed out by the manufacturer.

### Steps of drone operation and image capturing

This disaster management project utilised the RG101 drones for basic aerial surveys in unreachable areas, reducing the need for human intervention. It should be noticed that these drones are classified as toys and do not require a permit to operate. However, basic knowledge of calibration and health and safety measures for drone piloting and process mapping is necessary to identify areas where images are needed. The drones could cover an area of 5000 m^2^.

According to the drones’ instruction manuals, the first step included satellite signal acquisition: an open area was selected for launching to ensure sufficient GPS satellite signals. This was the assembly point for the students (see Figure 1; centre image between groups 1 and 5, community office and assembly point). The drone and remote control were powered on and paired, which was indicated by a sound signal. This was followed by the calibration process. Gyro calibration ensured flight stability by balancing the drone, while geomagnetic calibration helped the internal compass accurately respond to GPS signals. Both steps were confirmed with a ‘beep’ sound from the remote.

The third step involved the drone acquiring GPS signals for precise navigation, with a view displayed on a connected smartphone. This also allowed the return-to-home function to be activated if necessary. The fourth step comprised flight operations, during which basic flight manoeuvres were conducted, such as ascending, rotating and directional movements, while still maintaining the view from the connected smartphone. The one-key return feature was tested to ensure the drone could return autonomously in emergencies (Drone Play 2025).

### Ethical considerations

Ethical clearance to conduct this study was obtained from the Tshwane University of Technology Research Ethics Committee (No. REC2023-12-087).

## Results

The results of this empirical investigation suggested a community that has developed some resilience and means or abilities to cope with existing or dynamic pressures. As captured in Table 1, technical aspects and their indicators are further explored and rated as part of the holistic outlay of the community profile, where (√) = Observable or in-place; (n/a) = Not observed or measured and (-) = Not existent or Not in-place in terms of group observations (Table 1).

As reflected in Table 1, illegal electricity (Figure 3b) connections are rife in the community, while this fact is not farfetched from a community that is informal. The reported fatalities linked with this illegal practice continue to be challenging. The other great concern for the community is access to clean water every time. Evidently, the government continues to supply water during frequent times; the use of underground water (Figure 3a) adjacent to pit toilet facilities poses another great human health threat. Across the groups, the results suggest strong political and governance issues within the community, which goes well for the mobilisation and community awareness strategies. This is further emphasised by the strong reported communications and network settings within the community; these could have been indigenous or technological advantages, such as the ability to use social media groups as part of information sharing.

The below discussion captures the snapshot view of the community at large. It is thus important to note the interconnected nature of services required for the delivery of the key drivers of technical aspects of community services as articulated in the discussions.

## Discussion

This case study has highlighted the varying conditions of a typical informal settlement in South Africa. The study revealed poor infrastructure, poor political organisation and poor community governance, along with other significant triggers contributing to an undesirable community environment (Kolowa, Daams & Kuffer 2024). In this community, it appears that road infrastructure was at least considered during its establishment. This is similar to findings by Chavunduka and Chaorwa-Gaza (2021), which found that undocumented structures of law and order existed in those informal settlements.

The discussion is organised by priority, addressing the state of existing infrastructure, the improvement of community institutions, the community’s economy, human and social capital, and the maintenance of the natural ecosystem. These factors were rated as highly vulnerable and in need of more attention (Chavunduka & Chaonwa-Gaza 2021; Vallance & Rudkevitch 2021). There is clear evidence that, to some extent, the community is effectively self-governing and organised to address basic needs, such as upholding the rule of law and order. However, the presence of biculturalism or ethnocentrism was observed, reflecting a fear of the consequences of crime and criminal practices (Molho et al. 2024). This is similar to examples provided by Andreoli and Viola (2023) in modern society and progressive communities, where multicultural communities coexisted harmoniously and shared mutual respect.

While such cohesion is expected in a country such as South Africa, which promotes the policy of integration and cohabitation, these results were similar to findings by Milton and Chris’s (Milton 2023) investigation in New Zealand. It can be concluded that the existence of these religiously diverse groups could provide a solid foundation for emergency responses.

### Infrastructure capitals

The entire community relies on illegal electricity connections. Further examination of these findings indicates a potential crisis regarding electricity and energy supply within the community. Similar to other informal settlements, formalising this settlement would require more than just the establishment of infrastructure and electricity provisions. Water and sanitation supply needs are also critical (Geyevu & Mbandlwa 2022; Mensah 2022). Given the extent of illegal electricity connections, it is only a matter of time before incidents of electrical shocks and shack fires are recorded. It is plausible that these incidents are already occurring but are not reported to the authorities (Paulino & Paulino 2023). The benefits of the government rolling out these services would be substantial in the long run, potentially in the form of electricity billing and payment for basic services. While this point could be the main focus, it is essential to recognise that, as it stands, the municipality is losing a significant amount of revenue because of ongoing illegal electricity connections.

### Economic capitals

While the case study was conducted during the week, it was surprising to note the number of unemployed youths within the community. Although this was not quantitatively measured, the situation mirrors the national unemployment rate of 36% (Khalid et al. 2021). As a result, the community relies heavily on government grants, which is an observation similar to other regions of the country (Khalid et al. 2021). To combat levels of poverty, it was noticed that almost all households practised subsistence farming, a finding supported by research conducted by Mpundu and Bopape (2022). These conclusions align with several sustainable development goals, including poverty alleviation, zero hunger and good health and well-being.

While these acts of community resilience were observed, their sustainability is not guaranteed given the shortage of potable water. As noticed by Grangxabe et al. (2023), the community was similarly exposed to high levels of illegal waste dumping. While some forms of waste grouping and recycling were spotted in various households, the authors concluded that the situation created a conducive environment for pests and vector challenges. This was also observed as a form of income generation for some community members (Hidalgo-Crespo et al. 2023).

### Human and social capital

There is still much to be done regarding the human and social capacity of the community. Typical of a new establishment, the observation of the Tswaing community indicated that it was relatively young, with the oldest members likely not exceeding 55 years of age. This is consistent with trends observed in many informal settlements and the age and gender disparities often found in such communities in the Global South (Arce et al. 2021; Quesada-Román 2022). Given how the area was initially established, individuals via tribal authority had purchased plots that were then strategically allocated in almost equal sizes. This should aid in the processes of formalising the area, similar to findings by Gqomfa, Maphanga and Shale (2022) regarding the general formation of informal settlements.

Regarding human development and capacity building, it is important to understand that specific capacities are built on the foundation of existing resources (Bhagavathula et al. 2021). In this context, the City of Tshwane was observed to be working on building the community’s capacity for disaster awareness; however, the effectiveness of this initiative cannot be easily measured.

### Natural ecology capitals

The general landscape of the area is surrounded by wetlands, and the management of natural resources has been poor. This includes challenges in waste collection in an area that is informally established (Grangxabe et al. 2023). While this could be linked to a lack of knowledge and information regarding the preservation of natural resources, the general application of this view can be connected to the understanding of the role that natural resources, such as wetlands, play in a community. It is noticed that the area has a significant amount of underground water, making it sensible that many households would have a borehole on the same site (Figure 2). However, the dangers associated with this include the potential risks of contamination from the existence of long-drop toilets not far from the wells (Rao & Mogili 2022).

While these issues have been extensively investigated and documented by researchers such as Yahaya et al. (2023) in Nigeria, Binado, Kpieta and Amoah (2023) in Ghana and Molapo (2021) in South Africa, it is important to notice that the same region (Hammanskraal) has recently been battling a cholera outbreak (Makgopela & Radikonyana 2023; Obasa, Botes & Palk 2023). The authors of this case paper do not suggest the source of the previous outbreak but merely point out an existing situation that could spiral out of control if not carefully monitored. It should be observed that the government continues to provide potable water at strategically placed communal sites in the area. However, the assurance of continuous availability of this water cannot be guaranteed.

### Limitations

Several limitations exist in this study. No community interviews were conducted other than information provided by community leaders. While the circumstances were typical for an informal settlement, the generalisation may not apply in other informal areas. Also the data collected is representative of that particular time period.

## Conclusion

The findings present a compelling narrative for the need to conduct vulnerability assessments of communities. These assessments would not only assist with community preparation for eventualities but also help document necessary responses by the authorities involved in disaster response. This highlights the need for the development of a learning programme that integrates technology to assess community vulnerability and raise awareness of associated risks, ultimately enhancing community preparedness. The technology applied in this case study is basic in nature and incorporates community understanding and the ability to predict hazards that may pose a future threat to the studied community. This study explores successful strategies for community vulnerability assessments in environmental health and aims to bridge the gap between knowledge and informed decision-making to identify existing vulnerabilities and further estimate their impact on the community.

## References

[CIT0001] Andreoli, E. & Viola, P., 2023, ‘The multicultural state: Hypothesis for framing a concept’, *CALUMET* 18, 68–84.

[CIT0002] Arce, S.G., Jeanneret, C., Gales, J., Antonellis, D. & Vaiciulyte, S., 2021, ‘Human behaviour in informal settlement fires in Costa Rica’, *Safety Science* 142, 105384. 10.1016/j.ssci.2021.105384

[CIT0003] Berse, K.B., 2018, ‘Capacity assessment’, in F.C. Llanes (ed.), *Sakunang darating, saklolo’y tayo rin: Disaster risk reduction and management handbook for academic institutions*, pp. 133–158, University of the Philippines Press, Quezon City.

[CIT0004] Bhagavathula, S., Brundiers, K., Stauffacher, M. & Kay, B., 2021, ‘Fostering collaboration in city governments’ sustainability, emergency management and resilience work through competency-based capacity building’, *International Journal of Disaster Risk Reduction* 63, 102408. 10.1016/j.ijdrr.2021.102408

[CIT0005] Binado, T., Kpieta, A. & Amoah, S., 2023, ‘Conflicting necessities: Prefiguring pit latrine and quality of groundwater linkages in Ghanaian communities’, *International Journal of Energy and Water Resources* 7(2), 245–257. 10.1007/s42108-022-00222-z

[CIT0006] Burns, B. & Burns, M., 2013, *Wilderness GPS: A step-by-step guide (Mountaineering Outdoor Basics)*, Mountaineers Books, Seattle, WA.

[CIT0007] Cabello, V.M., Véliz, K.D., Moncada-Arce, A.M., Irarrázaval García-Huidobro, M. & Juillerat, F., 2021, ‘Disaster risk reduction education: Tensions and connections with sustainable development goals’, *Sustainability* 13(19), 10933. 10.3390/su131910933

[CIT0008] Chavunduka, C. & Chaonwa‑Gaza, M., 2021, ‘The political economy of urban informal settlements in Zimbabwe’, in A.R. Matamanda, V. Nel & I. Chirisa (eds.), *Urban geography in postcolonial Zimbabwe: Paradigms and perspectives for sustainable urban planning and governance*, pp. 287–305, Springer, Cham.

[CIT0009] Comes, T., Mayag, B. & Negre, E., 2014, ‘Decision support for disaster risk management: Integrating vulnerabilities into early-warning systems’, in C. Hanachi, F. Bénaben & F. Charoy (eds.), Proceedings of the Information systems for crisis response and management in Mediterranean countries: First International conference, ISCRAM‑med 2014, Springer International Publishing, Toulouse, October 15–17, 2014, vol. 196, pp. 178–191.

[CIT0010] Drone Play, 2025, *RG101 GPS drone 8K professional dual HD Camera*, viewed 22 April 2025, from https://droneplay.co.uk/.

[CIT0011] Flax, L.K., Jackson, R.W. & Stein, D.N., 2002, ‘Community vulnerability assessment tool methodology’, *Natural Hazards Review* 3(4), 163–176. 10.1061/(ASCE)1527-6988(2002)3:4(163)

[CIT0012] Gautam, D. & Khanal, S.C., 2009, *Community based disaster risk reduction: Contribution to Hyogo framework of action (Mercy Corps Nepal)*, Mercy Corps Nepal, Lalitpur.

[CIT0013] Geyevu, M. & Mbandlwa, Z., 2022, ‘Economic conditions that leads to illegal electricity connections at Quarry Road Informal Settlement in South Africa’, *International Journal of Special Education* 37(3), 11069–11078.

[CIT0014] Gqomfa, B., Maphanga, T. & Shale, K., 2022, ‘The impact of informal settlement on water quality of Diep River in Dunoon’, *Sustainable Water Resources Management* 8(1), 27. 10.1007/s40899-022-00629-w

[CIT0015] Grangxabe, X.S., Maphanga, T., Madonsela, B.S., Gqomfa, B., Phungela, T.T., Malakane, K.C. et al., 2023, ‘The escalation of informal settlement and the high levels of illegal dumping post-apartheid: Systematic review’, *Challenges* 14(3), 38. 10.3390/challe14030038

[CIT0016] Hidalgo-Crespo, J., Amaya-Rivas, J., Ribeiro, I., Soto, M., Riel, A. & Zwolinski, P., 2023, ‘Informal waste pickers in guayaquil: Recycling rates, environmental benefits, main barriers, and troubles’, *Heliyon* 9(9), e19775. 10.1016/j.heliyon.2023.e19775 37809813 PMC10559109

[CIT0017] Hou, J.Z., Hearn, G., Johnston, K.A., Villeneuve, M., Hebbani, A. & Carroll, M., 2025, ‘Embedding humanity in building sustainable community disaster resilience in Australia: A humanistic communication perspective’, *Australian Journal of Emergency Management* 40(1), 48–56. 10.47389/40.1.48

[CIT0018] Hung, K.K., Mashino, S., Chan, E.Y., MacDermot, M.K., Balsari, S., Ciottone, G.R. et al., 2021, ‘Health workforce development in health emergency and disaster risk management: The need for evidence-based recommendations’, *International Journal of Environmental Research and Public Health* 18(7), 3382. 10.3390/ijerph18073382 33805225 PMC8037083

[CIT0019] Imperiale, A.J. & Vanclay, F., 2016, ‘Experiencing local community resilience in action: Learning from post-disaster communities’, *Journal of Rural Studies* 47(pt. A), 204–219. 10.1016/j.jrurstud.2016.08.002

[CIT0020] Khalid, W., Akalpler, E., Khan, S. & Shah, N.H., 2021, ‘The relationship between unemployment and economic growth in South Africa: VAR analysis’, *Forman Journal of Economic Studies* 17(1), 1–32. 10.32368/FJES.20211701

[CIT0021] Kolowa, T.J., Daams, M.N. & Kuffer, M., 2024, ‘Do informal settlements contribute to sprawl in sub-Saharan African cities?’, *Sustainable Cities and Society* 113, 105663. 10.1016/j.scs.2024.105663

[CIT0022] Makgopela, P.S. & Radikonyana, P.S., 2023, ‘Drinkable and potable water in Hammanskraal, Tshwane Metropolitan Municipality: A service delivery challenge or a fallacy?’, *Journal of Public Administration* 58(2), 284–299. 10.53973/jopa.2023.58.2.a4

[CIT0023] Mbola, P., Nkosi, D.V. & Morakinyo, O.M., 2024, ‘Disaster management training for environmental health: A narrative literature review’, *Jàmbá: Journal of Disaster Risk Studies* 16(1), 1–9. 10.4102/jamba.v16i1.1706 PMC11447721 39363963

[CIT0024] Mensah, J.K., 2022, ‘Electricity and informal settlements: Towards achieving SDG 7 in developing countries’, *Energy Research & Social Science* 93, 102844. 10.1016/j.erss.2022.102844

[CIT0025] Milton, C., 2023, ‘Taking it with me: A South African’s cultural complex in Aotearoa New Zealand’, in T. Singer (ed.), *Cultural complexes in Australia: Placing psyche*, pp. 153–178, Routledge, London.

[CIT0026] Molapo, R.E., 2021, ‘Potential groundwater contamination from pit latrines in Ga‑Maja and Reefentse’, Master’s thesis, University of Pretoria, Pretoria.

[CIT0027] Molho, C., De Petrillo, F., Garfield, Z.H. & Slewe, S., 2024, ‘Cross-societal variation in norm enforcement systems’, *Philosophical Transactions of the Royal Society B* 379(1897), 20230034. 10.1098/rstb.2023.0034 PMC10799737 38244602

[CIT0028] Morgan, C.L., 2011, *Vulnerability assessments: A review of approaches [Monograph]*, IUCN, Gland.

[CIT0029] Mpundu, M. & Bopape, O., 2022, ‘Analysis of farming contribution to economic growth and poverty alleviation in the South African economy: A sustainable development goal approach’, *International Journal of Economics and Financial Issues* 12(5), 151–159. 10.32479/ijefi.13429

[CIT0030] Mubarak, A., Amiruddin, R. & Gaus, S., 2019, ‘The effectiveness of disaster prevention and mitigation training for the students in disaster prone areas’, *IOP Conference Series: Earth and Environmental Science* 235, 012055. 10.1088/1755-1315/235/1/012055

[CIT0031] Obasa, A., Botes, M. & Palk, A., 2023, ‘Collective responsibility during a cholera outbreak: The case of Hammanskraal’, *South African Journal of Bioethics and Law* 16(3), 99–104. 10.7196/SAJBL.2023.v16i3.1250

[CIT0032] Paulino, M. & Paulino, L.M., 2023, ‘Causes of household fires in rural areas: An exploratory research’, *Asian Journal of Community Services* 2(5), 389–394. 10.55927/ajcs.v2i5.4036

[CIT0033] Quesada-Román, A., 2022, ‘Disaster risk assessment of informal settlements in the Global South’, *Sustainability* 14(16), 10261. 10.3390/su141610261

[CIT0034] Rao, S.M. & Mogili, N.V., 2022, ‘Water management: Understanding the role of decentralized sanitation technologies such as pit toilets in environmental pollution’, in R. Brinkmann (ed.), *The Palgrave handbook of global sustainability*, pp. 1–23, Palgrave Macmillan, Cham.

[CIT0035] Sahar, S., Magda, S., Ruud, H. & Tuna, O., 2017, *National disaster risk assessment: Words into action guidelines – Governance system, methodologies and use of results*, United Nations Office for Disaster Risk Reduction, Geneva.

[CIT0036] Skybrary, 2025, *Monsoon influenced humid subtropical climate (Cwa)*, viewed 23 April 2025, from https://skybrary.aero/articles/monsoon-influenced-humid-subtropical-climate-cwa-0.

[CIT0037] South Africa, 2002, *Disaster management Act 57 of 2002*, Government Gazzete, viewed 21 April 2025, from https://www.gov.za/documents/disaster-management-act.

[CIT0038] Statistics S A, 2022, *Cencesus 2022*, Statistisc South Africa, viewed 23 January 2024, from https://www.statssa.gov.za/?page_id=4286&id=11353.

[CIT0039] United Nations Office for Disaster Risk Reduction (UNISDR), 2005, ‘Hyogo framework for action 2005–2015: Building the resilience of nations and communities to disasters’, in Proceedings of the World conference on disaster reduction, UNISDR, Geneva, January 18–22, 2005, pp. 1–25.

[CIT0040] United Nations, 2025, *The 17 sustainable development goals*, Department of Economic and Social Affairs Sustainable Development, viewed 22 April 2025, from https://sdgs.un.org/goals.

[CIT0041] Usman Kaoje, I., Abdul Rahman, M.Z., Idris, N.H., Razak, K.A., Wan Mohd Rani, W.N.M., Tam, T.H. et al., 2021, ‘Physical flood vulnerability assessment using geospatial indicator-based approach and participatory analytical hierarchy process: A case study in Kota bharu, Malaysia’, *Water* 13(13), 1786. 10.3390/w13131786

[CIT0042] Vallance, S. & Rudkevitch, A., 2021, ‘Post‑disaster recovery and social capital’, in D.V. McQueen (ed.), *Oxford research encyclopedia of global public health*, pp. 1–18, Oxford University Press, Oxford.

[CIT0043] Weichselgartner, J., 2001, ‘Disaster mitigation: The concept of vulnerability revisited’, *Disaster Prevention and Management: An International Journal* 10(2), 85–95. 10.1108/09653560110388609

[CIT0044] Whitney, M., 2014, *Vulnerability assessment methods (ASPIRES report)*, FHI 360, Washington, DC.

[CIT0045] World Health Organization (WHO), 2018, *The sphere handbook: Humanitarian charter and minimum standards in humanitarian response*, 4th edn., Geneva, viewed 04 December 2024, from https://www.spherestandards.org/handbook.

[CIT0046] Yahaya, T.O., Bashar, D.M., Liman, U.U., Umar, J.A., Abdulrahim, A. & Gomo, C.B., 2023, ‘Effects of Pit Latrines on borehole and well water in Maryland, Lagos, Nigeria’, *Journal of Advances in Environmental Health Research* 11(1), 20–27. 10.34172/jaehr.2023.03

[CIT0047] Zack, N., 2023, *Ethics for disaster*, 2nd edn., Rowman & Littlefield Publisher, Lanham, MD.

